# Acute Lower Lip Swelling: A Mere Anaphylactic Reaction or a Rare Abscess Location?

**DOI:** 10.7759/cureus.48971

**Published:** 2023-11-17

**Authors:** Anas Mahmoud, Lefika Bathobakae, Robert Giannetti, George Naaman, Joseph Affortunato

**Affiliations:** 1 Internal Medicine, St. Joseph’s University Medical Center, Paterson, USA; 2 Emergency Medicine, St. Joseph’s University Medical Center, Paterson, USA

**Keywords:** pott's puffy tumor, igg4 related sialadenitis, dental abscess, oral abscess, facial angioedema, allergy and anaphylaxis, methicillin resistant staphylococcus aureus (mrsa), hiv, lip abscess, lip swelling

## Abstract

An abscess is a collection of pus secondary to an immune response to a pathogen. It can occur anywhere in the body, with the skin as the most common organ involved. A lip abscess is a rare condition. Generally, it may be due to an infective agent such as a virus or bacteria entering through a skin wound, or through hematogenous spread when there is a severe underlying condition such as a general condition disorder or immunodeficiency. It requires hypervigilance during the examination and throughout the treatment course with intravenous antibiotic therapy and urgent surgical drainage, as it may cause significant complications regarding localization, lymphovascular drainage, and ultimate spread. Persistent abscess, necrotic tissue, or cavitated lesions are worrisome and it is important to rule out immunosuppression or a methicillin-resistant staphylococcus. In this case, a 22-year-old male patient with a unilateral lip abscess, misdiagnosed as an allergic food reaction, responded well to external drainage and antibiotic therapy.

## Introduction

Acute unilateral painful lip swelling is not a common condition and can carry high mortality if overlooked or left untreated [1]. Most commonly, lip swelling develops secondary to trauma or an allergic reaction, leading physicians to disregard a possible infectious etiology for this condition [2]. Infectious causes can be bacterial or viral, and the herpes virus should be suspected in the case of any unilateral painful swelling. Rapid swelling with discoloration can be a symptom of vascular tumors such as hemangioma and lymphangioma. Careful examination of oral mucosa for vesicles or ulcers is more likely to be a viral cause [3]. Staphylococcus aureus and streptococcus viridans are the leading bacterial causes of lip abscesses. Low immunity status in diabetes mellitus or HIV can predispose patients to develop infections. The main course of treatment is incision and drainage of the abscess and a proper course of antibiotics targeted against the causative organism. Our patient represents a unique case of severe lower lip swelling misdiagnosed as anaphylaxis, given the absurd swelling size of his lower lip, acuity of onset, absence of fever, and the patient’s history of a new type of food intake before his presentation.

## Case presentation

**Figure 1:**
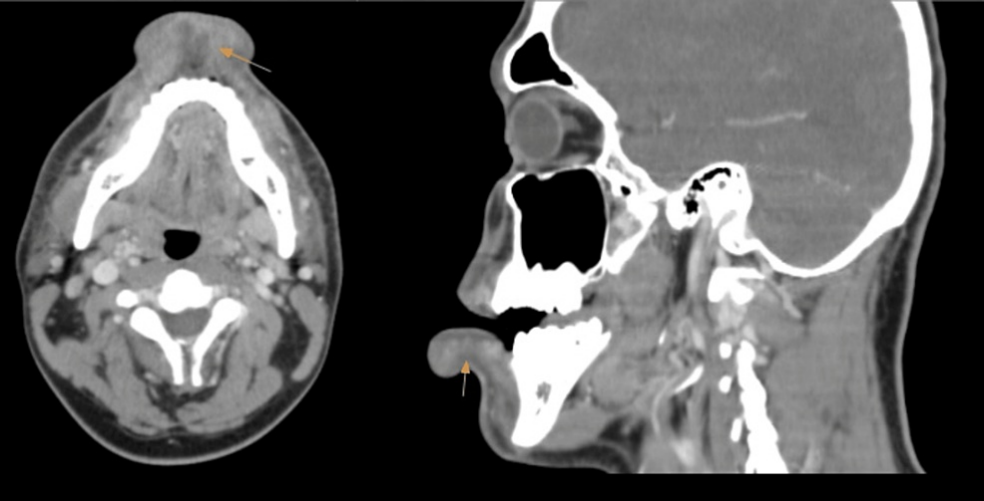
CT scan of the head and neck (sagittal on right side; axial on left side) There is a pronounced edema of the lower lip with a very limited edema of the upper lip. The associated peripherally enhancing collection extends to the margin of the gingiva (yellow arrows).

A 22-year-old male patient with a medical history of HIV (human immunodeficiency virus) presented at the emergency department seeking evaluation for lip swelling and general malaise. The patient reported that his symptoms began after trying an African dish for the first time, however, he could not remember its name or the ingredients used in its preparation. His symptoms progressively worsened and he experienced difficulty swallowing solid food, prompting him to seek medical attention at the ED. He denied having a history of allergic reactions. He also denied rash, itching, and shortness of breath. On examination, there was significant lower lip swelling; however, the oropharynx was clear, no tongue or upper lips swelling, and no signs of respiratory distress. A single dose of dexamethasone and epinephrine was given, and the patient was observed for a few hours and discharged safely. The patient returned the next day for persistent swelling of his lower lips. He had a blood pressure of 118/89 mmHg, a heart rate of 78 beats per minute, and a temperature of 36.9 °C. On examination, he had indurated lower lip swelling with a scant purulent discharge from an abscess with an opening on the inner mucosal lower lip, which was firm to the touch without a cystic sensation. Upon further questioning, the patient stated that he had a pimple on his chin and attempted to pop and squeeze it. Blood work showed WBCs of 5k, the last CD4 count 754 with low viral load on RT-PCR. The patient is compliant with HIV medication bictegravir/emtricitabine/tenofovir alafenamide and follows up regularly with an infectious disease specialist. CT scan of head and neck soft tissue showed a markedly pronounced edema of the lower lip, associated with a peripherally enhancing collection extending to the gingiva's margin (Figure 1). 

**Figure 2:**
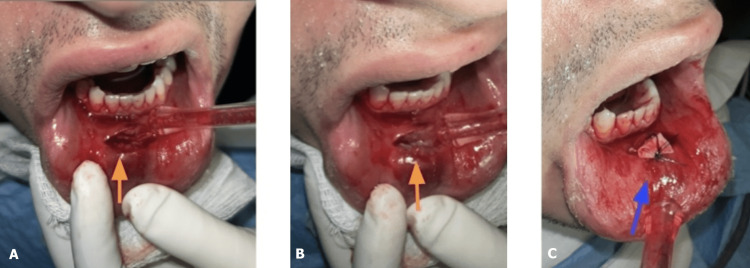
Incision and drainage of the lower lip abscess As indicated with yellow arrows: small yellow calcified calcite stones (A-B). Blue arrows: a drain was kept and the incision site was sutured (C).

Oral and maxillofacial surgery (OMFS) were consulted. Upon examination, it was decided to open, remove small yellow calcified calcite stones, drain the abscess, and keep a plastic drain to be removed two days later (Figure 2). Meanwhile, the tissues were sent for culture analysis. The patient was given one dose of intravenous ampicillin-sulbactam and discharged home on a week of amoxicillin-clavulanic acid. Cultures came back positive for methicillin-resistant Staphylococcus aureus (MRSA), which is sensitive to doxycycline, and the patient was contacted to change antibiotics. A week later, the patient presented later to the OMFS clinic for suture removal, and the swelling improved with the proper course of antibiotics. 

## Discussion

Acute swelling of lips occurs more commonly in anaphylactic reactions. An isolated lip abscess is a rare condition and is easily missed in clinical settings. It is more challenging to suspect a lip abscess without the five characteristic cardinals of acute inflammation (redness, heat, swelling, pain, and loss of function). Bacterial causes of lip swelling present more frequently as a skin abscess that penetrates inside or a primary mucosal abscess. Skin abscesses occur more frequently secondary to staphylococcus aureus or streptococcus. Primary mucosal abscesses can also be caused by anaerobic bacteria as well. Bacterial infections can cause swelling without abscess formation, mainly in patients with immunocompromised status. On the other hand, herpes, varicella, and other viruses commonly present with ulcers and vesicles, leading to lip swelling. Herpes zoster more commonly occurs as excruciating ulcers than swelling or redness. Isotretinoin, used in the treatment of acne vulgaris, can cause lip abscesses secondary to its dry mucous membranes effect and cheilitis that induce fissuring and cracking of the lips and consequently increases the odds of developing bacterial infection due to the breakdown of the mucocutaneous barrier [4]. Lip augmentation procedures can also result in lip swelling which can occur acutely or years later after the procedure [5]. 

Staphylococcus aureus, a Gram-positive, coagulase-positive cocci, is a commensal bacteria i.e. one that lives in different body organs such as skin, sebaceous glands, nose, and gut, causing no harm to healthy humans. Twenty percent of people are persistent nasal carriers of Staphylococcus aureus, making staph infection one of the most common infections overall. Hence, antibiotics have been targeting Staphylococcus aureus for ages, making the bacteria evolve and develop resistance. Methicillin-resistant Staphylococcus aureus (MRSA) is a widely common cause of infections in hospitalized patients and, unfortunately, has become a community-acquired infection, especially in patients with chronic immunodeficiency. Human immunodeficiency virus (HIV) is a leading cause for acquiring MRSA infections. Vancomycin is the drug of choice for MRSA infections, and developing resistance to vancomycin is gradually becoming a pandemic with the emergence of vancomycin-resistant staphylococcus aureus (VRSA) [6]. 

Weak immunity increases the risk of developing infections. Diabetes, innate and acquired human immunodeficiency, cancer, and immunosuppressive medications can lead to uncontrolled bacterial infections. It is worth knowing that HIV can disrupt the oral microflora, and this disturbance can lead to higher odds of developing oral infections [7]. HIV also increases the risk of developing bacterial infections in internal organs, like the pancreas [8]. Inflammatory bowel disease (IBD) can be complicated with variable oral manifestations such as mucogingivitis, deep linear ulcerations, or lip swelling with vertical fissures. Experiencing stress in addition to Herpes virus can increase the risk of developing cold sores which can lead to bacterial superinfection in some instances. 

A cross-sectional study of HIV patients has found a higher incidence of oral infections in HIV patients [9]. Peritonsillar abscesses are the most common deep infection of the head and neck, most commonly due to group A streptococcus (Streptococcus pyogenes), presenting as fever, malaise, sore throat, dysphagia, and hot potato muffled voice. Drainage and antibiotics are the mainstay of treatment [10]. A dental abscess (tooth abscess) occurs over months, usually secondary to dental decay, which takes a few months to approach the dental pulp. A periapical abscess develops at the tip of the root of the tooth and can extend to the maxillary bones and cause severe pain. Periodontal abscess, on the other hand, affects the bones that support the tooth, and it does occur with either gum diseases or periodontitis. Gingival abscess can develop in the gums secondary to trauma and low immune status. 

A submandibular abscess can extend to the pharyngeal spaces or to the floor of the mouth and cause swelling, commonly referred to as "Ludwig's angina." Odontogenic infections are polymicrobial, i.e., caused by aerobes or anaerobes, with the most common organisms being viridans streptococci. The common anaerobes are Fusobacterium spp. and Prevotella spp. Poorly controlled diabetes mellitus and immunodeficiency in cases of HIV or steroid intake make patients more prone to develop severe infections [10]. The sinus tract can present with simultaneous extraoral and intraoral abscess, manifesting as primary molar with chronic periapical abscess [11]. Sialadenitis refers to inflammation or infection of the salivary glands, with the most commonly affected ones being the parotid and submandibular glands. It can present as an acute bacterial suppurative infection or chronic changes with cysts and sinus tracts. IgG-4 disease can target salivary glands causing obstructive sialadenitis, and the biliary tract, causing obstruction [12]. Sjögren's syndrome can also present as granulomatous sialadenitis. Pott's puffy tumor is osteomyelitis of the skull bones, leading to forehead swelling. It occurs most commonly secondary to the direct spread of bacterial infection from the surrounding tissues, such as the nasal cavity, sinuses, or oral cavity [13]. Angular cheilitis, a common, non-contagious, inflammatory condition affecting the corners of the mouth or oral commissures can also develop in IBD or other conditions such as eczema, vitamin B deficiency, or excessive saliva.

Lip abscess can be detrimental as untreated abscess can lead to necrosis secondary to interruption of vascular supply. Direct spread to the brain or hematogenous spread to distant organs has been reported with lip abscesses, for instance, a case of pulmonary emboli and fatal necrotizing pneumonia occurred secondary to lip abscess due to MRSA infection in an immunocompetent patient [14]. Oral infections can also spread hematogenously, leading to infective endocarditis and other deep infections [15]. Brain abscess is not an uncommon target of direct spread of oral infections [16]. 

Angioedema is the main differential for bacterial causes of lip swelling. Angioedema is a sudden swelling of deep layers of the skin with fluids. Angioedema can affect any organ, but it more commonly affects the eyes, lips, and genitalia. The leading four causes are allergy, idiopathic, drug-induced, or hereditary. Allergies to food, insect bites, pollens, or radiocontrast media can be life-threatening as they can cause throat swelling and affect breathing [17]. Medications such as angiotensin-converting enzyme (ACE) inhibitors could cause angioedema due to a blockade of bradykinin degradation, which increases vascular permeability through substance P release, which in turn induces vasodilation and fluid extravasation; the required treatment is to stop the medication [18]. Penicillin, aspirin, and non-steroidal anti-inflammatory drugs like ibuprofen can also cause non-allergic drug-induced angioedema. Eosinophilia is associated with angioedema, characterized by fever and urticaria. Hereditary angioedema is more common in females and occurs more commonly in children due to a blood protein deficiency. On the contrary, acquired angioedema occurs later in life and is caused by C1 inhibitor deficiency in patients with B-cell lymphoma. Parvovirus, infectious mononucleosis, and helicobacter pylori can also cause angioedema [19]. 

## Conclusions

A lower lip abscess can be a life-threatening condition if left ignored and/or untreated. Detailed history-taking with specific questions about skin lumps, dental, or lip manipulation should be asked. History of immunodeficiency should raise suspicion for acquiring MRSA infections and should be treated with broader antibiotics. Appropriate consultation with oral and maxillofacial surgeons should be sought as incision and complete drainage is the mainstay treatment. Head and neck imaging with CT scans should be taken as infection can spread to more serious deeper tissues and ultimately the brain. Follow-up is as important as treatment given the dangerous location of the lip abscess. 

## References

[1] Tekcan Şanlı DE, Boyacı Z (2021). Immunocompetent young patient presenting with unilateral lip abscess due to peeling exfoliated lip skin. Turk Arch Otorhinolaryngol.

[2] Amin D, Satishchandran S, Drew S, Abramowicz S (2021). Diagnosis and treatment of lip infections. J Oral Maxillofac Surg.

[3] Alciato L, Bonfils P, Rubin F (2019). Unilateral oral, pharyngeal and laryngeal vesicles. Eur Ann Otorhinolaryngol Head Neck Dis.

[4] Huoh KC, Chang KW (2013). Lip abscess associated with isotretinoin treatment of acne vulgaris. JAMA Dermatol.

[5] Scarano A, Di Carmine MS, Greco Lucchina A (2023). Chronic lip edema and pain secondary to lip augmentation procedure: histological, scanning electron microscopy and X-ray microanalysis evaluation. Eur Rev Med Pharmacol Sci.

[6] Lakhundi S, Zhang K (2018). Methicillin-resistant Staphylococcus aureus: molecular characterization, evolution, and epidemiology. Clin Microbiol Rev.

[7] Sadagopan A, Mahmoud A, Begg M (2023). Understanding the role of the gut microbiome in diabetes and therapeutics targeting leaky gut: a systematic review. Cureus.

[8] Rajkumar K, Mahmoud A, Abdalla M, Grossman M (2023). Pancreatic abscess: an infection occurring with minimal tissue present. Case Reports in Clinical Medicine.

[9] Mohamed N, Saddki N, Yusoff A, Mat Jelani A (2017). Association among oral symptoms, oral health-related quality of life, and health-related quality of life in a sample of adults living with HIV/AIDS in Malaysia. BMC Oral Health.

[10] Galioto NJ (2008). Peritonsillar abscess. Am Fam Physician.

[11] Bashar AK, Akter K, Chaudhary GK, Rahman A (2019). Primary molar with chronic periapical abscess showing atypical presentation of simultaneous extraoral and intraoral sinus tract with multiple stomata. BMJ Case Rep.

[12] Mahmoud A, Mohamed A, Alyassin N, Grossman M, Cavanagh Y (2023). Cholangiocarcinoma, primary sclerosing cholangitis, or IgG4-sclerosing cholangitis: similar presentations with different managements. Case Reports in Clinical Medicine.

[13] Mahmoud A, Mekheal E, Varghese V, Michael P (2022). Can a cerebral congenital anomaly present in adulthood?. Cureus.

[14] Bruno GJ, Bruno JM, Miyake AA (2007). Community-acquired methicillin-resistant Staphylococcus aureus infection with fatal necrotizing pneumonia from lip abscess: a case report. J Oral Maxillofac Surg.

[15] Mahmoud A, Khalid AB, Ehle A, Kahf Y, Afzal A, Peltzer B (2023). Does heart failure mask Candida tricuspid endocarditis?. Cureus.

[16] Karageorgiou I, Chandler C, Whyte MB (2014). Silent diabetes mellitus, periodontitis and a new case of thalamic abscess. BMJ Case Rep.

[17] Brown T, Gonzalez J, Monteleone C (2017). Angiotensin-converting enzyme inhibitor-induced angioedema: A review of the literature. J Clin Hypertens (Greenwich).

[18] Kaplan AP, Greaves MW (2005). Angioedema. J Am Acad Dermatol.

[19] Gunatilake SS, Wimalaratna H (2014). Angioedema as the first presentation of B-cell non-Hodgkin lymphoma--an unusual case with normal C1 esterase inhibitor level: a case report. BMC Res Notes.

